# Sociodemographic Variables in Offender and Non-Offender Patients Diagnosed with Schizophrenia Spectrum Disorders—An Explorative Analysis Using Machine Learning

**DOI:** 10.3390/healthcare12171699

**Published:** 2024-08-26

**Authors:** Andreas B. Hofmann, Marc Dörner, Lena Machetanz, Johannes Kirchebner

**Affiliations:** 1 Adult Psychiatry and Psychotherapy, University Hospital of Psychiatry Zurich, Faculty of Medicine, University of Zurich, 8006 Zurich, Switzerland; lena.machetanz@pukzh.ch; 2 Department of Consultation-Liaison-Psychiatry and Psychosomatic Medicine, University Hospital Zurich, University of Zurich, 8091 Zurich, Switzerland; marc.doerner@usz.ch; 3 German Center for Neurodegenerative Diseases (DZNE) within the Helmholtz Association, 39120 Magdeburg, Germany; 4 Forensic Psychiatry and Psychotherapy, University Hospital of Psychiatry Zurich, Faculty of Medicine, University of Zurich, 8006 Zurich, Switzerland; johannes.kirchebner@pukzh.ch

**Keywords:** machine learning, artificial intelligence, forensic psychiatry, schizophrenia spectrum disorders, offenders, advanced statistics, psychotic disorders, sociodemographic variables

## Abstract

With the growing availability of medical data and the enhanced performance of computers, new opportunities for data analysis in research are emerging. One of these modern approaches is machine learning (ML), an advanced form of statistics broadly defined as the application of complex algorithms. ML provides innovative methods for detecting patterns in complex datasets. This enables the identification of correlations or the prediction of specific events. These capabilities are especially valuable for multifactorial phenomena, such as those found in mental health and forensic psychiatry. ML also allows for the quantification of the quality of the emerging statistical model. The present study aims to examine various sociodemographic variables in order to detect differences in a sample of 370 offender patients and 370 non-offender patients, all with schizophrenia spectrum disorders, through discriminative model building using ML. In total, 48 variables were tested. Out of seven algorithms, gradient boosting emerged as the most suitable for the dataset. The discriminative model finally included three variables (regarding country of birth, residence status, and educational status) and yielded an area under the curve (AUC) of 0.65, meaning that the statistical discrimination of offender and non-offender patients based purely on the sociodemographic variables is rather poor.

## 1. Introduction

Due to the increasing amount of medical data and the advances in digitalization, there is a growing need for statistical approaches with the ability to comprehensively and efficiently analyze said data. One of these approaches is machine learning (ML). Machine learning is an evolving branch of complex algorithms designed to mimic human intelligence by learning from the environment. As an example of artificial intelligence, ML algorithms learn from and adapt their performance to the raw data fed to them [1,2]. The “learning” can be understood as the process of finding patterns in a dataset, helping to uncover new information without going through hypothesis testing [3]. Following technical progress in processing power, the algorithms can easily be applied to large data sets while keeping computing times at acceptable levels [4]. This allows the analysis of not only a large quantity of variables but also their interplay, which makes ML highly suitable for the evaluation of multifactorial outcomes [5,6].

In psychiatric research, statistics mostly rely on null hypothesis significance testing (NHST) or regression models, presenting a simple representation of the relationship between an independent and a dependent variable [7]. However, these methods have certain shortcomings. (1) To avoid an accumulation of alpha error, only a limited number of variables can be analyzed, restricting the possibility of investigating multifactorial outcomes [8]. (2) NHST, for example, is not able to analyze the interplay of variables, impairing the possibility of investigating complex relationships and phenomena [4]. (3) As the statistical approach is selected in advance, it may not fit the data structure. (4) In accordance with the principle of falsification in NHST, null hypotheses can only either be falsified—meaning that the alternative hypothesis is assumed—or not; however, this is not, in turn, the verification of the null hypothesis [9]. This requires the research question to be precisely and unambiguously defined in advance, which limits explorative investigations for undetected patterns in data. Repeatedly, despite its legitimation, NHST, as the “default statistical practice” [10], has come under increasing criticism as it does not accommodate all types of research undertakings [11,12,13,14].

Mental disorders are generally influenced by a variety of factors and their interplay, instead of being monocausal and linear. For instance, schizophrenia may develop in individuals with (a) a certain vulnerability and (b) a certain set of conditions [15,16]. The same is true for outcomes and events during the course of a mental disorder, e.g., aggression [17]. Oversimplification in the understanding of mental disorders has been identified as a conceptual crisis in psychiatric research [18]. Keeping the limitations of widely used statistical approaches outlined above in mind, it becomes clear that they may not be suitable when investigating multidimensional constructs as they are presented in psychiatry. Here, the qualities of ML offer new opportunities. Apart from their ability to detect patterns in large datasets and analyze complex, non-linear interrelations, ML algorithms can also help to evaluate the quality of a statistical model, e.g., with receiver operating characteristics (ROCs), area under the curve (AUC), sensitivity, or specificity [4]. This quantification of a predictive model allows a transparent evaluation of the soundness of the model [19].

So far, ML is still rarely used for research purposes in forensic psychiatry in general, and if so, mostly for the prediction of violence [20]. Existing work mainly focuses on risk assessment, while rather little fundamental research is conducted [21,22]. This poses a major issue since it is vital to understand the differences between patients diagnosed with schizophrenia spectrum disorders (SSD)who commit crimes and those who do not. Recent research performed by our group investigated the similarities and differences of these patients in various aspects, e.g., aggression and suicidal behavior [23,24]. So far, there is no comprehensive ML analysis of solely sociodemographic factors. Filling this gap in knowledge might help to prevent patients from becoming perpetrators at all.

The role of sociodemographic factors in the development of criminal behavior has been repeatedly discussed, e.g., low economic status, low achievement in school, or social isolation [25,26,27]. Therefore, a potential association between sociodemographic variables and criminal behavior shall be investigated. While individuals with SSDs have an elevated risk of expressing criminal behavior, the majority of affected patients do not come into conflict with the law but rather are at higher risk of victimization compared to the general public [28,29,30,31]. With the expression of criminal behavior in mental disorders being under-researched, it seems sensible to further evaluate possible contributors and protective factors to close this research gap. Regarding statistical procedures, ML outperforms common techniques due to various reasons, as described above. We therefore opted to use the means of ML due to greater flexibility and the lack of need for a priori assumptions [32].

The following study aims to outline the procedures, benefits, and limitations of ML in psychiatric research in a paradigmatic research question with the objective of discriminating between offenders and non-offenders with SSDs based solely on sociodemographic variables. To our knowledge, this study is the first one to follow this goal.

## 2. Materials and Methods

### 2.1. Study Population

The total sample comprised a study group of offender patients (OP, n = 370) and a comparison group of non-offender patients (NOP, n = 370), all of whom were diagnosed with SSDs according to ICD-9 or ICD-10 (chapters F20.0 to F25.9) [33,34]. Both groups were matched by gender. To evaluate whether comparability between the two was feasible, basic sample characteristics such as country of birth, marital status, and diagnosis, as well as comorbidities and aggressive behavior, were assessed.

#### 2.1.1. Forensic Psychiatric Subpopulation (OP)

The OP sample (n = 370) stemmed from patients admitted to the Centre for Inpatient Forensic Therapy at the University Hospital of Psychiatry Zurich between 1982 and 2016, with the majority being admitted after the year 2000. This institution, being the largest forensic psychiatric inpatient treatment facility in German-speaking Switzerland, serves two purposes: patients are either admitted by court order for reduction of their risk of reoffending through treatment of their underlying psychiatric illness or they are referred from penitentiary settings for the treatment of acute psychiatric syndromes. OP patients were convicted because of violent (e.g., homicide, physical or sexual assault, and arson) and non-violent reasons (e.g., threatening behavior, crimes against property, and violation of traffic, drug, and firearm regulations).

#### 2.1.2. General Psychiatric Subpopulation (NOP)

The NOP (n = 370) sample stemmed from patients admitted for general psychiatric inpatient treatment at the Centre for Integrative Psychiatry of the University Hospital of Psychiatry Zurich. Amongst other specialized wards, the facility focuses on the subacute treatment of psychotic disorders, usually for 6 to 8 weeks or longer if needed.

### 2.2. Data Source and Extraction

Data were retrospectively assessed based on the patients’ medical files. The case files were rather comprehensive and included extensive information on the referenced hospitalization, including reports by various medical healthcare professionals and reports on previous in- and outpatient treatment. For the OP group, the files also included testimonies, police reports, court proceedings, and information regarding the course of previous imprisonments and detentions. Data assessment and extraction were performed through directed qualitative content analysis by two experienced psychiatrists according to a rating protocol based on a set of criteria originally described by Seifert and Nedopil and adapted under the supervision of experienced forensic and general psychiatric researchers and clinicians [35]. To evaluate for inter-rater reliability, a random subsample of 10% of all cases was independently encoded by another researcher. With a Cohen’s Kappa of 0.78, inter-rater reliability was considered substantial [36].

### 2.3. Selection of Predictor Variables

Since the purpose of our research presented here was to determine whether—and if so, which—sociodemographic factors divide the NOP group from the OP group, 48 items from the following domains were selected as predictor variables: age, gender, country of birth, status of residency, profession of faith, marital status and close family, living situation at the time of admission to the referenced hospitalization, highest school-leaving certificate, learned profession and employment status at the time of admission to the referenced hospitalization, type of legal guardian during childhood and adolescence, and membership in social associations as a measure of social integration. We consider this selection appropriate since these variables often play an important role in public discussions about criminal development and—despite the retrospective study design—are still collectible without influencing data quality. For a detailed list of all predictor variables and their precise definitions, please refer to Appendix A.

### 2.4. Data Analysis Using Machine Learning

We used supervised ML to uncover the most significant variables that distinguish the OP group from the NOP group among a large set of parameters, selecting the model with the highest predictive power. Unlike unsupervised ML, which is employed to uncover hidden patterns in datasets without a defined outcome variable, supervised ML trains algorithms on labeled datasets and uses these algorithms to predict specific outcomes—in this case, “OP: true” vs. “OP: false” [37].

**Figure 1:**
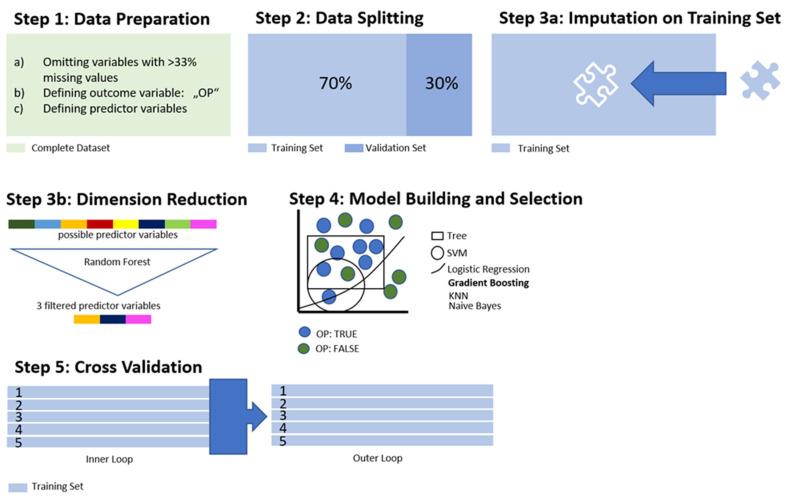
Data processing and training of the algorithm. Legend: OP = Offender patients; SVM = support vector machines; KNN = k-nearest neighbors; SVM = support.

Figure 1 provides an overview of the statistical steps, which are further detailed below. All steps were carried out using R version 3.6.3 (R Project, Vienna, Austria) and the MLR package v2.171 (Bischl, Munich, Germany [38]). R and the MLR package are common and easy-to-use software with reliable and replicable codes. In particular, the MLR package allows for many ML operations, including variable reduction and imputation. Calculations for the confidence intervals of the balanced accuracy were performed with MATLAB R2019a (MATLAB and Statistics Toolbox Release 2012, The MathWorks, Inc., Natick, MA, USA; License obtained via the University of Zurich) using the add-on “computing the posterior balanced accuracy” v1.0.

#### 2.4.1. Preprocessing

Categorical variables were transformed into binary code, whereas continuous and ordinal variables remained unadjusted. The outcome variable was categorized as either “OP: true” or “OP: false”, with the latter being defined as the positive class in further analyses (Figure 1, Step 1). In ML, the algorithm or model needs to be trained before it can be applied to new data [39]. To provide the algorithm with data from which it could learn to detect patterns, our data set was split into one training set, comprising 70% of all cases, and a validation set with the remaining 30% of all cases, which was stored aside and remained untouched for the following process (Figure 1, Step 2).

To allow the inclusion of the total population and to avoid omissions resulting in an increased risk of bias, the imputation of missing values was carried out [40]. We employed mean imputation for numerical variables and mode imputation for categorical variables due to their simplicity and efficiency. Mean imputation helps preserve the central tendency of the data, reducing biases in the mean structure. Mode imputation maintains the distribution of the most frequent categories, ensuring the integrity of categorical data distributions. To apply the same coefficients in the imputation of missing values in the validation set, we created an “ImputationDesc” object with the coefficients used in the imputation on the training set (see Figure 1, Step 3a). The ImputationDesc object stores all relevant information about the imputation and can be used to impute the test data set the same way as the training data.

A primary goal of this study was to identify the key variables among the 48 possible ones. However, as data are mostly nonlinear and nonparametric, there was a high chance of overfitting, a common obstacle in ML. In practice, overfitting refers to the algorithm learning too well from the training data, with random fluctuations in the data being picked up by the model [41]. We conducted variable reduction using a random forest algorithm (Figure 1, Step 3b). Initially, all available variables were included. Iteratively, the least important variables, as determined by random forest importance scores, were removed. The reduction process continued until the addition of another variable did not improve the AUC by more than 5%. This approach ensured that only the most predictive variables were retained, optimizing the model’s performance and interpretability. This resulted in 3 predictor variables (see Section 3) and also helped to prevent extensive computing times. Thus, the preprocessing was concluded.

#### 2.4.2. Training of the Algorithm

Seven algorithms—logistic regression, decision trees, random forest, gradient boosting, k-nearest neighbor (KNN), support vector machines (SVMs), and naïve Bayes—were applied to the training set for building discriminative models from multiple perspectives and to ensure the robustness of our results. Logistic regression and naïve Bayes provide straightforward, interpretable models, while decision trees and random forest offer flexibility and resistance to overfitting. Gradient boosting and SVMs are powerful for handling complex, non-linear relationships, and KNN is suitable for capturing local data structures. In employing multiple algorithms, the authors aimed to facilitate the comprehensive validation of findings and increase confidence in the results by using cross-verifying patterns identified by different methods [42,43]. The algorithms were assessed according to the parameters listed in Table 1. The model with the best performance was then selected for model validation on the validation set (Figure 1, Step 4).

As discussed above, avoiding overfitting was crucial in the process. To reduce the risk beyond dimensionality reduction, we conducted cross-validation on the training set in the form of nested resampling [45]. The entire data processing and model training process was conducted with cross-validation, and the models’ performance was tested in an outer loop also embedded in cross-validation. This approach allowed us to artificially create different subsamples of the same dataset while keeping the validation subset untouched. (Figure 1, Step 5).

#### 2.4.3. Validation of the Algorithm

**Figure 2:**
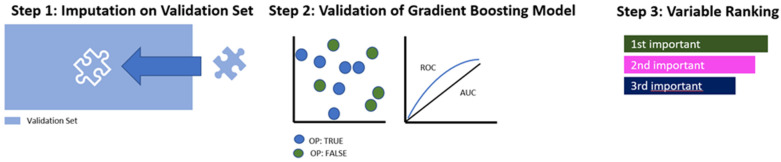
Model building and testing the performance on the validation dataset. Legend: OP = Offender patients; AUC = area under the curve (level of discrimination); ROC = receiver operating characteristic curve.

The following steps were all performed on the validation set (30% of the total population), which had remained strictly untouched by the procedures described in steps 3–5. Imputation of missing values was carried out in the same manner as on the training set, with the imputation weights previously saved (Figure 2, Step 1).

The most suitable model, which had been identified in Figure 1, Step 4, was applied and evaluated in terms of its performance parameters (Figure 2, Step 2).

In the last step, the identified predictor variables were ranked in accordance with their relative influence within the selected model (Figure 2, Step 3).

## 3. Results

The basic characteristics of our sample, which were evaluated to check comparability between OP and NOP samples, showed a similar distribution of age and gender, as well as psychiatric main diagnosis, with the majority of patients suffering from paranoid schizophrenia. Regarding psychiatric comorbidities, OP patients showed a higher prevalence of personality disorders and substance use disorders (Table 2).

Out of the seven algorithms applied in the model building process, gradient boosting showed the best performance parameters on the training set, yet the AUC only yielded 0.69 (Table 3). With a sensitivity of 77%, the algorithm identified nearly ¾ of all NOP samples correctly. At the same time, the best-performing algorithm was able to identify only half of all OP samples correctly.

Out of all possible 48 predictors/discriminative variables (see Appendix A for the full list), the following remained after the reduction of dimensionality through random forest: Switzerland as country of birth, illegal residency in Switzerland, and failure to complete compulsory schooling (Table 4). Adding another item to the model did not significantly improve the AUC, meaning that the three variables were more indicative of discrimination between the groups than all other variables.

**Figure 3:**
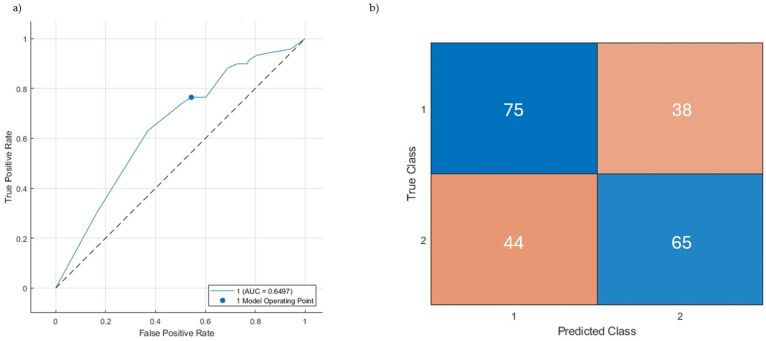
The final model’s AUC curve (**a**) and confusion matrix (**b**).

After having applied the gradient boosting model to the validation set, both sensitivity and specificity yielded 63%. The AUC was 0.65 (95% confidence interval 0.58–0.72), which was slightly lower than on the training set (Table 5). Figure 3 displays the ROC curve (a) and the confusion matrix (b), displaying the numbers of true positives/negatives and false positives/negatives of the final model.

**Figure 4:**
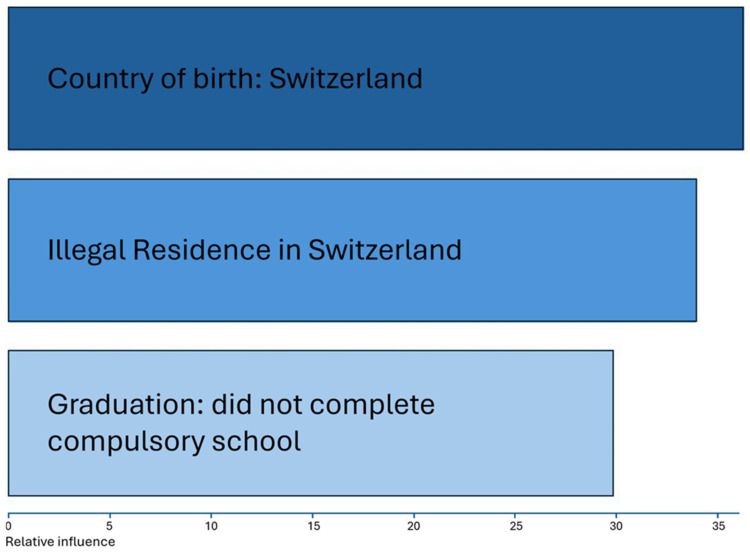
Ranking of predictor variables in accordance with their importance in the model (through gradient boosting).

The variables contributed differently to the model: country of birth (Switzerland) emerged as the most influential, closely followed by illegal residence in Switzerland and failure to complete compulsory schooling (see Figure 4).

## 4. Discussion

By using supervised ML, we were able to analyze whether sociodemographic characteristics are powerful discriminative variables between offenders and non-offenders with schizophrenia spectrum disorders. As outlined above, the application of ML is useful when analyzing complex phenomena with intertwining variables, such as criminal behavior, which is considered to be driven by multiple factors. A similar analysis with NHST, which is widely applied to psychiatric research regardless of whether it is actually suitable for the research question, would not have been feasible, as it does not allow the analysis of the interplay of multiple variables, and the number of variables in this analysis would have led to an accumulation of alpha error. Furthermore, supervised ML offers the possibility of exploring data without the need to define the hypothesis in advance, which allows the discovery of unknown patterns in data. This makes ML especially suitable for exploratory analyses in areas characterized by scarcity in the pre-existing literature, which can be used to form a sound and distinct hypothesis. The presented study aims to serve as an example of ML as a powerful data-driven tool to analyze a wealth of complex data, detecting new patterns and thus offering new possibilities for research off the beaten path of widely applied traditional statistical methods.

While the current study focused on the presentation of an exemplary application of a machine learning-based methodology in psychiatric research, we would like to discuss the results on a clinical level. Regarding the baseline parameters of both study groups, the OP group had a higher proportion of comorbid substance use disorders. This finding corresponds to previous research stating that patients suffering from SSDs are more likely to commit violent crimes in cases of comorbid substance abuse [28,29].

In our model, “country of birth: Switzerland” and “illegal residence in Switzerland” emerged to be the most and second-most influential, respectively. OP patients were less frequently born in Switzerland and simultaneously resided more often without a legal basis. In other words, a history of migration turned out to be highly important when distinguishing between offending and non-offending SSD patients. While the personal experience of migration has been shown to be a risk factor for developing psychotic disorders [46,47,48], the differences regarding criminal behavior are not easy to explain. In a sample of forensic patients in Denmark, the proportion of migrants committing violent crimes exceeded that of individuals of Danish ethnicity. This finding was attributed to the elevated risk of psychosis in migration and the underlying linkage of schizophrenia and violent behavior [49]. A population study from Sweden covering a span of nearly 40 years found that both male and female schizophrenia patients not born in Sweden were at higher risk of committing violent acts, although the factor “Born abroad” turned out to be only one among several others, such as substance abuse or past violence [50]. Several research works covering immigration and crime found no or only weak associations (either positive or negative), but these studies did not investigate violence in the context of psychiatric disorders in general and SSD in particular. In addition, no subgroup analyses regarding the social status of migrants, as well as the country and culture of origin, were conducted [51,52,53]. Transferability to the results of this study is therefore impaired.

Moreover, illegal residency might result in barriers regarding access to healthcare [54,55]. Although all individuals residing in Switzerland, undocumented immigrants included, can receive mental health care upon request [56], disadvantages in daily life cannot be ruled out as illegal residents might fear denunciation to authorities and thus decline medical consultation or may not be aware of available health care structures. In addition, treatment is often limited to essential procedures in cases of emergency; thus, access to all potentially available services is not granted [57]. Consequently, existing psychiatric or emerging disorders are possibly at higher risk of exacerbation.

In general, schizophrenia is associated with reduced academic achievements, and affected individuals are less likely to attain higher educational levels [58]. In our study, OP patients showed a higher prevalence of failure to conclude compulsory schooling (26%; NOP: 5.6%). The Swedish-based population study previously mentioned found higher rates of violent crimes in male schizophrenic patients with low intelligence quotient [50], which is a risk factor for dropping out of school [59]. In our study, IQ was not tested, and furthermore, the higher rate of failure in forensic patients is probably caused by multiple factors. In addition to the factors mentioned above, migration while school-aged impairs school attendance and performance [60], and, depending on the country of origin, individuals potentially face scarcely developed educational systems. Lastly, the high rate of failure may also be attributed to a higher burden of disease.

The performance metrics of our Gradient Boosting Model indicate only poor to moderate efficacy. The Balanced Accuracy of 63.1% suggests a fair balance in identifying both positive and negative cases, as sensitivity and specificity perform similarly. An AUC of 0.65 reflects poor discriminatory power [61]. The PPV of 66.4% and NPV of 59.6% indicate the model’s moderate predictive reliability [62]. Importantly, the NPV with a confidence interval of 49.8% to 68.8% is the least significant, since the lower threshold of the CI is below 50%. This implies that there is substantial uncertainty around the NPV estimate, and in some cases, the NPV might be less than 50%, meaning that the model’s ability to correctly identify true negatives could be as bad as random guessing.

The finding that the model’s performance was rather poor sparks hope for clinicians, as it shows that variables regarding the patient’s sociodemographic background, which mostly cannot be therapeutically targeted due to their biographical nature, are not, on their own, determinants of criminal development as a negative outcome during the course of SSDs.

That said, both groups are more similar than one could have expected. The present AUC and the balanced accuracy should therefore not be recognized as weak statistical performance parameters, but rather indicate that the OP and NOP groups have many sociodemographic features in common.

A crucial step is the separation of training and validation data, which allows the model to face an unbiased sample, or, as Matthew Carbone called it, a “litmus test” for the performance of the model on new data [63]. However, this requires both sets of data to be of the same type, meaning that it needs to be of the same distribution. In general, keeping in mind that ML is a data-driven approach, the algorithms’ performance is as good as the data provided [64]. In our case, there are some limitations to our data that need to be addressed, the first being the retrospective data extraction bias, in which data quality is not comparable to a prospectively standardized study, resulting in decreased robustness [65]. Additionally, various unspecific variables that could influence the course of treatment cannot be measured in a scientifically sound way in retrospective studies. This, for example, applies to important states such as perceived loneliness. However, with lengths of stay of up to several years in court-mandated inpatient treatment, forensic psychiatric research faces the problem of collecting large enough samples for robust results, which is why we opted for the retrospective approach regardless of its limitations. This brings us to the second caveat in data quality—while the sample of 740 can be considered large from a forensic psychiatric point of view, it is rather small for ML purposes. The smaller the sample, the less training data are fed to the algorithm, and the lower its statistical power [66]. This again stresses the fact that ML is ideal for analyzing big data but is not suitable for very small sample sizes. These also increase the risk of unequally distributed training and validation datasets. Therefore, the results obtained in this study should be reproduced in a multicenter project in order to collect a sufficient amount of data.

Regarding further methodological limitations, overfitting, a common issue in ML, has to be discussed. The term overfitting refers to a model incorporating noise in the training data to the extent that a high error rate occurs on new data, reducing its applicability and generalizability [67]. However, there are statistical steps to counteract the effect of overfitting, such as the cross-validation applied in this case [41]. By creating five random subsamples of our data and always using one as a validation set and the other four as training sets, error estimation is averaged over all five trials (folds), reducing variance and bias. While it is possible to perform cross-validation with even more folds, this also increases computing time and can be rather time-consuming depending on the technical prerequisites available. Data quality might also be impaired by missing values. In our study, all variables with more than 33% missing observations were omitted. The missing values of the remaining variables were then imputed, a potentially problematic but still recommended approach in order to maintain data quality [68,69]. Lastly, it is vital to consider various ethical challenges associated with the use of artificial intelligence in the field of forensic psychiatry, e.g., racial or gender bias [70].

Recently, a growing discussion about Explainable Artificial Intelligence (XAI) has emerged. XAI fosters transparency and understanding of machine learning models and advocates a human understanding of AI-generated models. Only then is it possible to determine when to trust the AI and when the AI should be distrusted, which is especially important in sensitive fields such as forensic psychiatry [71,72]. In our study, XAI might help clarify how and why the particular sociodemographic variables influence the model’s predictions. While our research focuses on identifying these variables to improve the care of non-European migrants in general and forensic settings, XAI helps to ensure that the model’s decisions are transparent and free from bias, thereby preventing further stigmatization of this vulnerable group. Additionally, XAI could provide insights into the moderate predictive power of our model. Future projects should therefore focus on incorporating XAI more thoroughly.

## 5. Conclusions

Merely sociodemographic attributes are not sufficient to explain the differences between general and forensic psychiatric patients suffering from schizophrenia spectrum disorders, as indicated by our model’s mediocre performance parameters. What appears to be negative is actually a glimmer of hope. In contrast to the variables described previously, a variety of other features can be influenced by clinicians, e.g., psychopathology, integration into social communities, and comorbid substance abuse.

Supervised Machine Learning facilitates multiple chances to explore undetected patterns within complex datasets. In particular, the under-researched field of forensic psychiatry benefits from these modern and increasingly available methods.

## Figures and Tables

**Table 1:** Brief explanation of included statistical parameters used in machine learning [44].

Performance Measures	Explanation
Receiver operating characteristics, area under the curve (AUC)	Overall ability of a model to discriminate between two groups, as indicated by graph plotting sensitivity and 1-specificity. The higher the AUC, the better the model distinguishes between positive and negative classes.
Balanced Accuracy	The average of sensitivity and specificity, providing a single measure that allows for interpreting both false positives and false negatives.
Sensitivity	The ability of a model to correctly identify true positives. Sensitivity is also called recall or the true positive rate.
Specificity	The ability of a model to correctly identify true negatives, also called the true negative rate.
Positive predictive value (PPV)	The proportion of positive test results that are true positives. Used to interpret an individual’s actual probability of being a true positive in case of a positive test result.
Negative predictive value (NPV)	The proportion of negative test results that are true negatives. Used to interpret an individual’s actual probability of being a true negative in case of a negative test result.

**Table 2:** Basic sample characteristics.

Variable Description	OP n/N (%)	Mean (SD)	NOP n/N (%)	Mean (SD)
Age at admission		34.2 (10.2)		36.2 (12.2)
Sex *: male	339/370 (91.6)		339/370 (91.6)	
Country of birth: Switzerland	167/370 (45.1)		245/367 (66.8)	
Marital status: Single	297/364 (81.6)		282/364 (77.5)	
Diagnosis: Schizophrenia	294/370 (79.5)		287/370 (77.6)	
Co-Diagnosis: Addiction Disorder	269/200 (72.9)		183/327 (56)	
Co-Diagnosis: Personality Disorder	47/370 (12.7)		26/370 (7)	

Legend: SD = Standard deviation; OP = Offender patients; NOP = Non-offender patients; n = subgroup with characteristics; N = total study population; * according to patients’ case files.

**Table 3:** Applied Machine Learning models and their performance in nested cross-validation.

Statistical Procedure	Balanced Accuracy (%)	AUC	Sensitivity (%)	Specificity (%)	PPV (%)	NPV (%)
Logistic Regression	62.20	0.68	74.90	49.50	58.20	69.60
Tree	63.00	0.64	82.30	43.80	57.70	74.00
Random Forest	62.4	0.68	78.3	46.5	57.9	73.4
**Gradient** **Boosting**	**63.3**	**0.69**	**76.7**	**49.9**	**59.3**	**72.1**
KNN	56.9	0.59	78.9	35	44.6	85.9
SVM	61.9	0.68	73.8	49.9	58.1	68.9
Naive Bayes	62.6	0.68	62.3	62.9	61.2	65.3

Legend: AUC = area under the curve (level of discrimination); PPV = positive predictive value; NPV = negative predictive value; KNN = k-nearest neighbors; SVM = support vector machines. Bold font highlights the algorithm with the best performance parameters.

**Table 4:** Absolute and relative distribution of relevant predictor variables.

Variable Description	OP n/N (%)	NOP n/N (%)
Country of birth: Switzerland	167/370 (45.1)	245/367 (66.8)
Illegal residence in Switzerland	95/370 (25.7)	34/367 (9.3)
Graduation: did not complete compulsory schooling	89/342 (26)	18/321 (5.6)

Legend: OP = Offender patients; NOP = Non-offender patients; n = subgroup with characteristics; N = total study population.

**Table 5:** Final performance measures of the gradient boosting model on validation data.

Performance Measures	% (95% CI)
AUC	0.65 (0.58–0.72)
Balanced Accuracy	63.1 (56.5–69.1)
Sensitivity	63 (53.6–71.6)
Specificity	63.1 (53–72.2)
PPV	66.4 (56.8–74.8)
NPV	59.6 (49.8–68.8)

Legend: AUC = area under the curve (level of discrimination); PPV = positive predictive value; NPV = negative predictive value; CI = Confidence interval.

## Data Availability

The following supporting information can be downloaded at: https://www.researchgate.net/publication/363044110_Coding_protocol_Pathways_into_delinquency_in_offenders_suffering_from_schizophrenia_spectrum_disorders?_sg%5B0%5D=-ouv9n-TZGVYwLNzNA2bbNtfTtmeQNdhhSQ-LYjjNDxDMyLjcB4vguIZNi9ybfdR_-BWL5X_anlM8pT205mWVZhQ0SQHmrjOyBkLTY4C.CsUdT-XjnI56I52UGGdJCVu5RF529xxsJdoeMgg6niRGe915la0jtcoQq1bpRUMolGL8TNd8w6wt3rMM3mDZpA&_tp=eyJjb250ZXh0Ijp7ImZpcnN0UGFnZSI6InByb2ZpbGUiLCJwYWdlIjoicHJvZmlsZSIsInByZXZpb3VzUGFnZSI6InByb2ZpbGUiLCJwb3NpdGlvbiI6InBhZ2VDb250ZW50In19 (accessed on 22 August 2024). The datasets generated and analyzed during the current study, as well as a detailed list of all our variables (including definitions and references), are available from the corresponding author on request.

## Appendix A

**Variable Code**	**Variable Label**
y (Outcome variable)	Is the patient forensic?
SD1	Age at admission?
SD2	Sex according to patient files
SD3a	Country of birth: Switzerland?
SD3b	Country of birth: Balkan region country?
SD3c	Country of birth: other European country?
SD3d	Country of birth: Middle east?
SD3e	Country of birth: Africa?
SD3f	Country of birth: other country?
SD3g	If 3b–3f do not apply, legal residence in Switzerland?
SD4a	Christian faith?
SD4b	Islamic faith?
SD5a	Marital Status (at time of the investigated offence)—married?
SD5b	Marital Status (at time of the investigated offence)—single?
SD6a	Living situation (at time of the investigated offence)—mental health care institution
SD6b	Living situation (at time of the investigated offence)—assisted living
SD6c	Living situation (at time of the investigated offence)—home alone
SD6d	Living situation (at time of the investigated offence)—home with others
SD6e	Living situation (at time of the investigated offence)—at parents’ home
SD6f	Living situation (at time of the investigated offence)—with relatives
SD6g	Living situation (at time of the investigated offence)—homeless
SD6h	Living situation (at time of the investigated offence)—prison
SD6i	Living situation (at time of the investigated offence)—other
SD7a	Highest graduation (at time of the investigated offence)—no compulsory schooling
SD7b	Highest graduation: (at time of the investigated offence)—compulsory schooling
SD7c	Highest graduation (at time of the investigated offence)—graduation
SD7d	Highest graduation (at time of the investigated offence)—college/university
SD8a	Learned profession: no apprenticeship
SD8b	Learned profession: college/university degree
SD8c	Learned profession: official/civil servant
SD8d	Learned profession: mercantile job
SD8e	Learned profession: non-mercantile job
SD8f	Learned profession: crafting job
SD8g	Learned profession: other job
SD9	Is the patient a nonworker (at time of the investigated offence)?
SD11	Is the patient a nonworker (majority of occupational time)?
SD12a	Rank at job: basal (majority of occupational time)?
SD12b	Rank at job: complex (majority of occupational time)?
SD14	Own children?
SD15	Any siblings?
SD17	Was the legal guardian married?
SD18a	Who was/is the legal guardian—birth parents?
SD18b	Who was/is the legal guardian—single parents?
SD18c	Who was/is the legal guardian—step-parents?
SD18d	Who was/is the legal guardian—one step-parent?
SD18f	Who was/is the legal guardian—grandparents?
SD18g	Who was/is the legal guardian—foster parents?
SD18h	Who was/is the legal guardian—child home?
SD19	Member in a (leisure) club?
